# The digital physician illusion: individual innovativeness, unlike artificial intelligence attitudes, drives rational drug use through health literacy

**DOI:** 10.3389/fpubh.2026.1958038

**Published:** 2026-10-06

**Authors:** Kaan Raif, Kemal Cek, Saime Ulucayli, Fazilet Aksu

**Affiliations:** 1 Department of Healthcare Organizations Management, Faculty of Health Sciences, Near East University, Nicosia, Türkiye; 2 Department of Accounting and Finance, Cyprus International University, Nicosia, Türkiye; 3 Department of Healthcare Organizations Management, Faculty of Economics and Administrative Sciences, Cyprus International University, Nicosia, Türkiye; 4 Department of Medical Pharmacology, Faculty of Medicine, DESAM Research Institute, Near East University, Nicosia, Cyprus

**Keywords:** artificial intelligence, digital health, health literacy, individual innovativeness, rational drug use, structural equation Modeling

## Abstract

**Background:**

The rapid integration of artificial intelligence into healthcare has transformed how individuals access medical information. However, the impact of artificial intelligence attitudes and individual innovativeness on clinically disciplined behaviors, such as rational drug use, and the mediating role of health literacy remains underexplored.

**Objective:**

This study aims to empirically investigate the structural relationships between artificial intelligence attitude, individual innovativeness, health literacy, and rational drug use within the digital health ecosystem of Northern Cyprus.

**Methods:**

A cross-sectional survey design was employed to collect data from 445 adults. The proposed relationships, integrating the Technology Acceptance Model and the Health Belief Model, were tested using Covariance-Based Structural Equation Modeling (CB-SEM) via IBM SPSS AMOS. A 5,000-resample bootstrapping procedure was utilized for mediation analysis.

**Results:**

The structural model demonstrated an acceptable fit to the data (*χ*^2^/df = 2.45, CFI = 0.935, TLI = 0.921, RMSEA = 0.062, SRMR = 0.058). The structural model revealed that individual innovativeness has a significant and positive impact on both health literacy and rational drug use. While a favorable attitude toward artificial intelligence significantly improves health literacy, it does not directly translate into rational drug use. Furthermore, the mediation analysis demonstrated that health literacy significantly mediates the relationship between individual innovativeness and rational drug use; however, no such mediating effect was observed between artificial intelligence attitude and rational drug use.

**Conclusion:**

Although positive attitudes toward artificial intelligence enhance health literacy by improving access to information, this cognitive advantage does not inherently foster the clinical discipline required for rational drug use. The findings highlight the “Digital Physician Illusion,” emphasizing that algorithmic guidance cannot replace the indispensable oversight of healthcare professionals.

## 1 Introduction

The rapid advancements in information and communication technologies have fundamentally transformed the delivery of healthcare services and the interactions between patients and healthcare professionals. This transformation has accelerated the shift from traditional hospital-centric models to patient-centric models where the patient plays an active role. Individuals now widely utilize not only healthcare professionals, but also artificial intelligence (AI) powered decision support systems, symptom checkers, chatbots, and mobile health applications to monitor their health status, assess symptoms, and explore treatment options (1, 2). Many generative AI systems offer an interaction experience that surpasses traditional web search by making personalized health information more accessible, thereby reinforcing individuals’ adoption of digital health technologies (3).

Individuals’ inclination toward digital health technologies and their adoption processes of new tools are closely related to the concept of individual innovativeness. This concept is defined as a psychological predisposition or personality trait toward the acceptance of new ideas, applications, and technologies (4, 5). According to Rogers’ diffusion of innovations theory, individuals are divided into five categories: innovators, early adopters, early majority, late majority, and laggards (6). In particular, individuals with a high level of innovativeness show lower resistance to uncertainty and exhibit more positive attitudes toward medical AI applications (4). In line with the relationship noted in the literature, individual innovativeness has been identified as a significant factor, whether direct or indirect in shaping readiness and willingness to engage with AI tools in healthcare and clinical education contexts (5, 7).

This wave of digitalization in healthcare has broadened the scope of health literacy (HL), transforming it into a multi-dimensional concept. Traditionally, HL was defined as an individual’s capacity to acquire, understand, and use health-related information. However, in today’s digital ecosystem, this definition also encompasses the competencies to access information in digital environments, critically evaluate it, and assess the accuracy of AI outputs (8, 9). Although general health literacy can be conceptualized broadly enough to include competencies related to digital and AI-related health literacy, it is still essential to distinguish operationally between these terms. General health literacy encompasses the foundational cognitive and social skills necessary to access, understand, appraise, and apply health information for daily medical decision making (10, 11). Digital health literacy (eHealth literacy) on the other hand, builds upon this foundation by defining the ability to seek, locate, comprehend, and evaluate health information from digital platforms and to apply this knowledge to address health-related problems (12, 13). AI-related health literacy, also referred to as algorithmic health literacy, is an emerging and specialized competency involving interaction with generative AI tools and large language models, critical evaluation of algorithmic recommendations, and identification of potential AI errors or ‘hallucinations’ (8, 14, 15).

While AI-based systems have the potential to simplify complex medical terminology and facilitate access to information, they also carry the risk of producing misleading information, sometimes referred to as “hallucinations” (14, 16). Individuals with low HL tend to misinterpret information in digital environments, apply algorithms without verifying their accuracy, and over-rely on online content. This creates serious health risks at both individual and societal levels (9).

These risks are most clearly manifested in the challenges of rational drug use (RDU). The World Health Organization defines RDU as individuals being able to obtain the appropriate drug, for the appropriate duration and dosage, according to their clinical needs and individual characteristics, easily and at the lowest cost for both themselves and their communities (17). However, recommendations from AI-based applications are increasing the tendency of patients to make their own decisions about drug use without consulting healthcare professionals (18). Especially in individuals with low HL, this situation leads to irrational behaviors such as inappropriate antibiotic use and non-compliance with treatment regimens, triggering serious health problems such as the development of antimicrobial resistance, adverse drug reactions, morbidity, mortality, and increased treatment costs (19, 20). In addition to this, irrational drug use poses serious threats not only to human health but also to environmental sustainability, as pharmaceutical residues lead to water, soil, and air pollution, biodiversity loss, ecotoxicity, and contribute to climate change (21–23). Conversely, high levels of HL and self-efficacy positively influence treatment adherence and drug safety by enabling patients to better understand their treatment plans. Indeed, HL plays a decisive and mediating role in transforming information obtained from digital health tools into RDU behavior (4, 24).

Previous research such as (4, 5) has systematically examined the determinants of artificial intelligence acceptance in healthcare, with a primary focus on clinicians’ and patients’ adoption intentions and perceived ease of use. Simultaneously, public health scholarship has identified health literacy as a significant predictor of medication adherence (10, 25) and self-medication behaviors (24, 26). Despite this progress, these two areas remain separate in both theory and research. Technology acceptance studies often neglect the translation of positive technological attitudes into clinically regulated patient behaviors. In contrast, clinical adherence models rarely consider the influence of rapidly emerging generative AI tools that may facilitate unregulated self-medication (18). Notably, prior research have not explained the structural pathway connecting intrinsic personality traits, such as individual innovativeness, and technological affinity, such as attitudes toward AI, to the complex construct of RDU, which requires more rigorous clinical oversight than simple compliance or avoidance of self-medication.

This study aims to fill this gap by empirically examining the disconnect between technological optimism and actual clinical compliance. Specifically we aim to determine the impact of individuals’ attitudes toward AI and their levels of innovativeness on their drug use behaviors. Another important objective is to empirically test the mediating role of HL in this complex relationship.

By establishing this novel structural model within the context of Northern Cyprus—as illustrated in Figure 1—the research not only provides a theoretical contribution to the global literature but also offers valuable empirical insights for the local health sector. Furthermore, selecting Northern Cyprus as the study setting offers unique contextual advantages. First, its isolated island geography minimises confounding cross-border dynamics. Second, its dense higher education network provides a highly innovative, technologically inclined population, which is ideal for examining the cognitive disconnect between digital technological optimism and actual clinical compliance. Finally, its pharmacy-centric healthcare system makes community pharmacies the primary point of direct patient contact, where foundational health literacy is uniquely critical as a cognitive gatekeeper to prevent unregulated, AI-facilitated self-medication.

**Figure 1:**
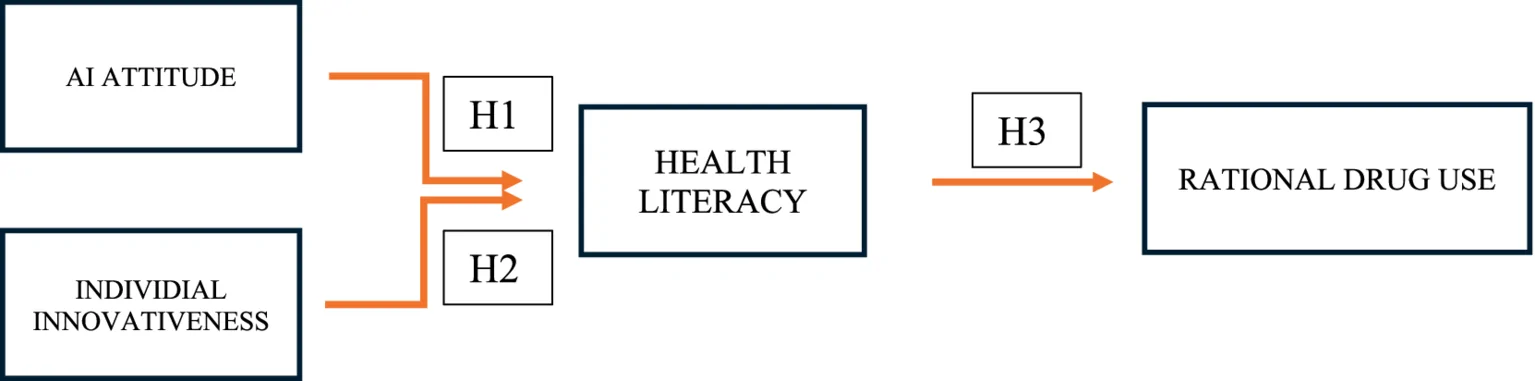
Conceptual framework of the study. Structural equation model with four variables: AI Attitude (top left), Individual Innovativeness (bottom left), Health Literacy (center), and Rational Drug Use (right). Orange arrows indicate: H1 from AI Attitude to Health Literacy; H2 from Individual Innovativeness to Health Literacy; H3 from Health Literacy to Rational Drug Use. Indirect effects H4 and H5 via Health Literacy are represented by the mediating pathway.

To explain the structural pathways of our conceptual model, we integrate the theoretical anchors of the Technology Acceptance Model (TAM) (27) and the Health Belief Model (HBM) (28). In the classical TAM, technology adoption is driven by perceived utility and perceived ease of use, which collectively shape user attitudes and behavioral intentions. Building upon this theoretical foundation, although the proposed model does not directly measure core TAM constructs such as perceived utility and perceived ease of use, it builds upon the core structure of this model by examining individual innovativeness and AI attitudes as critical exogenous determinants of technology acceptance. In line with established literature, individual traits and attitudes toward new technologies reflect the psychological drivers that shape people’s perceptions of usefulness and ease of use in digital health (29, 30). Specifically, a favorable attitude toward AI is expected to minimize cognitive barriers and simplify complex medical terminology, acting as an affective gateway that directly enhances an individual’s health literacy capacity.

H1: A favorable attitude toward artificial intelligence has a significant and positive effect on health literacy.

In parallel, within the HBM framework, health protecting actions are governed by cognitive dimensions such as perceived disease threat and self-efficacy (28). In this study, health literacy is positioned as the fundamental cognitive driver of these health beliefs, providing individuals with the cognitive self-efficacy required to accurately analyze medical risks and make informed clinical decisions. Highly innovative individuals, characterized by an inherent psychological predisposition toward novel technologies, exhibit proactive information-seeking behaviors, prompting them to actively use digital tools to dynamically enrich their health literacy capacity (12, 13).

H2: Individual innovativeness has a significant and positive impact on health literacy.

Consequently, health literacy serves as the primary cognitive gatekeeper in medication management, enabling patients to critically appraise health risks and directly fostering RDU by strengthening self-efficacy regarding treatment plans (10, 26).

H3: Health literacy has a significant and positive effect on rational drug use.

Ultimately, health literacy acts as the critical mediating bridge that tames technological optimism. It translates technological attitudes derived from the TAM and personal traits such as individual innovativeness into disciplined, safe clinical behaviors grounded in the HBM, thereby preventing unregulated self-medication driven by algorithmic hallucinations and automation biases (31, 32).

H4: Health literacy plays a mediating role in the relationship between artificial intelligence attitude and rational drug use.

H5: Health literacy plays a mediating role in the relationship between individual innovativeness and rational drug use.

## 2 Materials and methods

### 2.1 Research design and participants

This study employs a cross-sectional research design to empirically assess attitudes toward AI, individual innovativeness, and RDU, with HL analyzed as a mediating variable. The target population consists of adults (aged 18 and older) residing in the Northern Cyprus. To ensure the accuracy of the sampling framework, technical support was obtained from the Northern Cyprus Statistical Institute. According to the Institute’s 2025 end-of-year projections, the total population is 502,000 (54% male, 46% female), while the eligible target population (aged 18 and over) comprises 391,000 individuals (213,000 males and 178,000 females).

Data collection was conducted between May and July 2026 through a hybrid approach, incorporating both online surveys hosted on digital platforms and in-person fieldwork. To recruit participants online, the survey link was distributed across major social media networks and instant messaging applications such as Facebook community groups, WhatsApp, and LinkedIn. For the in-person fieldwork, potential respondents were approached in high-traffic public areas like university campuses, community centers, and commercial areas in all six districts (Nicosia, Famagusta, Kyrenia, Morphou, Trikomo, and Lefka) of Northern Cyprus.

To ensure eligibility, a mandatory screening question was placed at the beginning of the survey, requiring participants to confirm that they were at least 18 years of age and currently residing in Northern Cyprus. Utilizing a convenience sampling method across online and community settings, approximately 553 individuals were approached or invited to participate. A total of 476 responses were initiated, and following rigorous data cleaning to exclude incomplete, patterned, or invalid forms, a final cohort of 445 fully completed and valid responses was obtained, representing an effective response rate of approximately 80.5%. Although convenience sampling does not support population-wide inferential confidence intervals, this sample size (*n* = 445) comfortably exceeds the general recommendations and statistical power thresholds for covariance-based structural equation modeling (CB-SEM), easily meeting the established sample adequacy criteria outlined by Hair et al. (33) and Sekaran and Bougie (34).

### 2.2 Data collection tools and scales

Research data were collected using a questionnaire with five sections. The first section, the Demographic Information Section, aims to determine the basic characteristics of participants, including age, gender, marital status, education level, frequency of pharmacy visits, and presence of chronic diseases.

The remaining four sections of the questionnaire contain standardized scales designed to measure the study’s main variables. All of these scales use 5-point Likert-type ratings to capture participants’ opinions. The scales appear as follows:

- Attitude Towards AI: The 4-item “Attitude Towards AI Scale,” originally developed by Grassini (35) and adapted into Turkish by Arslan et al. (36), was used to assess participants’ attitudes toward AI.
- HL: The 6-item “European Health Literacy Scale Short Form (HLS-EU)”, originally developed by Pelikan et al. (11) and adapted into Turkish by Yeşildal (37), was used to measure participants’ level of HL.
- Individual Innovativeness: The “Individual Innovativeness Scale (IIS),” consisting of 20 items, originally developed by Hurt et al. (38) and adapted by Kılıçer and Odabaşı (39), was used to evaluate participants’ degree of individual innovativeness.
- RDU: The “RDU Awareness Scale in Adults,” developed by Aktaş and Selvi (40) and consisting of 15 items, was used to assess participants’ awareness regarding RDU.

To assess the reliability of the data collection instruments, the internal consistency of the scales was evaluated using Cronbach’s alpha. The analysis confirmed high reliability across all instruments within the current sample, yielding highly satisfactory *α* coefficients.

### 2.3 Analysis of data

Descriptive statistics and preliminary analyses were conducted using IBM SPSS Statistics. To test the proposed hypotheses, Covariance-Based Structural Equation Modeling (CB-SEM) was employed using IBM SPSS AMOS. The measurement model was first evaluated through Confirmatory Factor Analysis (CFA) to assess reliability, convergent validity, and discriminant validity. Subsequently, the structural model was tested using the Maximum Likelihood (ML) estimation method. Furthermore, to evaluate the mediating effects, a bootstrapping procedure with 5,000 resamples was applied to generate 95% bias-corrected confidence intervals (Table 1).

**Table 1:** Data collection scales.

Scale	Number of items	Original developer(s)	Turkish adaptation
Attitude Towards AI Scale	4	Grassini (35)	Arslan et al. (36)
European Health Literacy Scale Short Form (HLS-EU)	6	Pelikan et al. (11)	Yeşildal (37)
Individual Innovativeness Scale (IIS)	20	Hurt et al. (38)	Kılıçer and Odabaşı (39)
Rational Drug Use Awareness Scale in Adults	15	Aktaş and Selvi (40)	Aktaş and Selvi (40)

Summary of four measurement scales used for data collection: Attitude Towards AI Scale (4 items); European Health Literacy Scale Short Form HLS-EU (6 items); Individual Innovativeness Scale IIS (20 items); Rational Drug Use Awareness Scale in Adults (15 items).

### 2.4 Ethical considerations

The research strictly adhered to scientific ethical standards. Formal ethical approval was granted by the Near East University Scientific Research Ethics Committee (Approval Number: YDU/2026/144-2117, Date of approval: 21/05/2026). Prior to data collection, all participants were provided with detailed information regarding the study’s purpose, confidentiality guarantees, data storage procedures, and the voluntary nature of their participation, after which informed consent was obtained.

### 2.5 Use of artificial intelligence

During the preparation of this work, the authors utilized Google Gemini and Google NotebookLM solely for the purpose of literature synthesis, initial background research, and refining the narrative flow during the drafting of the Introduction and Literature Review sections. These AI-assisted tools were not used to conduct statistical analysis, interpret research findings, or generate empirical data. After using these tools, the authors reviewed, critically evaluated, and edited the content as necessary and take full responsibility for the final content and accuracy of the published article.

## 3 Results

Table 2 presents the demographic profile of the participants. The sample consists of a majority of female respondents (58.9%, *n* = 262) compared to males (41.1%, *n* = 183). Most participants are married (59.8%), while 27.4% are single. Regarding educational attainment, the largest group holds an undergraduate degree (43.4%), followed by those with graduate degrees (28.8%) and primary or high school education (27.8%). Additionally, the majority of respondents reported having no chronic diseases (69.4%), whereas 30.6% reported having at least one chronic condition. The average age of the participants was 41.17. Regarding the age distribution, 21.6% (*n* = 96) of the respondents were between 18 and 30 years old, 40.0% (*n* = 178) were between 31 and 45 years old, 35.7% (*n* = 159) were between 46 and 60 years old, and the remaining 2.7% (*n* = 12) were 61 years of age or older.

**Table 2:** Demographic characteristics of the participants.

Variables	Category	Frequency (*n*)	Percentage (%)
Gender	Male (0)	183	41.1
	Female (1)	262	58.9
Age	Average age	41.17	
Marital status	Single (0)	122	27.4
	Married (1)	266	59.8
	Other (2, 3)	57	12.8
Education level	Primary-High School (0–3)	124	27.8
	Undergraduate (4)	193	43.4
	Graduate (5)	128	28.8
Chronic disease	No (0)	309	69.4
	Yes (1)	136	30.6

To examine variation in scale scores across demographic groups, we conducted independent-samples *t*-tests and one-way ANOVA. The findings showed that male participants had significantly more positive attitudes toward Artificial Intelligence (AI) than female participants (*t* = 2.088, *p* = 0.037). This aligns with prior literature suggesting higher technology adoption readiness among males. Furthermore, participants without chronic illnesses demonstrated significantly higher AI adoption scores (*t* = 1.981, *p* = 0.048), which may be attributed to younger mean age and lower healthcare anxiety in non-chronic populations. Crucially, education level emerged as a universal differentiator across all construct dimensions *p* < 0.05. Post-hoc analyses indicated that higher education levels (undergraduate and postgraduate) significantly enhance Health Literacy, Individual Innovativeness, Rational Drug Use, and AI Acceptance. Higher educational attainment equips individuals with stronger information-evaluation skills, leading to greater readiness for AI integration and more conscious health-related decision-making, such as rational medication adherence.

Table 3 presents the descriptive statistics for the study’s main constructs. The findings indicate that the participants exhibit relatively high levels of RDU awareness (M = 3.77, SD = 0.53) and HL (M = 3.61, SD = 0.59). Similarly, attitudes toward AI (M = 3.60, SD = 0.80) and individual innovativeness (M = 3.48, SD = 0.51) are also above the midpoint of the 5-point Likert scale, indicating a generally positive orientation toward these constructs in the sample.

**Table 3:** Descriptive statistics, validity and reliability.

Construct	Mean	Std. deviation	Min	Max	CR	AVE
RDU	3.77	0.53	2.5	5	0.81	0.68
HL	3.61	0.59	1.67	5	0.86	0.70
AI attitude	3.6	0.8	1	5	0.78	0.65
Individual innovativeness	3.48	0.51	1.26	5	0.80	0.73

The reliability and validity of the measurement model were assessed using Confirmatory Factor Analysis (CFA). Internal consistency was established as the Cronbach’s alpha coefficients for all constructs exceeded the recommended threshold of 0.70. Furthermore, Construct Reliability (CR) and Average Variance Extracted (AVE) were evaluated to confirm convergent validity. All item loadings were significant, and the calculated CR values exceeded 0.70, while the AVE values exceeded the 0.50 threshold, indicating adequate convergent validity.

The internal consistency and reliability of the refined measurement scales were evaluated using Cronbach’s alpha (*α*), with results summarized in Table 4. All four constructs demonstrated high psychometric integrity, comfortably exceeding the standard academic reliability threshold of *α* > 0.70. Following scale purification via Confirmatory Factor Analysis (CFA), the retained item sets exhibited strong internal cohesion: AI Attitude (4 items, *α* = 0.879), Individual Innovativeness (9 items, *α* = 0.885), and Rational Drug Use (RDU) (7 items, *α* = 0.915) all achieved excellent reliability levels (*α* > 0.85). Additionally, the Health Literacy (HL) scale (6 items, *α* = 0.715) maintained acceptable reliability. These results confirm that deleting weak items during factor purification did not compromise scale stability; rather, it preserved robust construct reliability while optimizing the measurement instruments for structural model estimation.

**Table 4:** Internal consistency and reliability of the scales.

Construct	Number of items	Cronbach’s alpha (*α*)	Reliability level
AI Attitude	4	0.879	Excellent
Individual innovativeness	9	0.885	Excellent
RDU	7	0.915	Excellent
HL	6	0.715	Acceptable

Table 5 details the item factor loadings obtained from the Confirmatory Factor Analysis (CFA). All standardized factor loadings for the measurement items are statistically significant and well above the acceptable threshold of 0.50, ranging from 0.559 to 0.898. This indicates that the observed variables adequately capture their respective latent constructs, providing initial empirical evidence of the measurement model’s convergent validity. During the Confirmatory Factor Analysis (CFA), eight items from the RDU Scale and 11 items from the Individual Innovativeness Scale were excluded from the measurement model due to low standardized factor loadings (*λ* < 0.50) to achieve an acceptable model fit. We examined corrected item-total correlations and removed items with weak correlations (*r* < 0.30) with the scale score to ensure domain homogeneity. Modification indices and residual covariances were evaluated to identify and remove items with substantial cross-loadings or redundant error terms, thereby ensuring scale unidimensionality and structural elegance.

**Table 5:** Confirmatory factor analysis (CFA) item loadings.

Item no.	Scale items	Standardized factor loadings
1	I believe that AI will improve my life.	0.898
2	I believe that AI will improve my work.	0.877
3	I think I will use AI technology in the future.	0.833
4	I think AI technology is positive for humanity.	0.821

Item no.	Scale items	Standardized factor loadings
1	Find out about activities that are good for your mental well-being	0.704
2	Find information on how to manage mental health problems like stress or depression	0.692
3	Understand information in the media on how to get healthier	0.676
4	Judge if the information on health risks in the media is reliable	0.658
5	Judge when you may need to get a second opinion from another doctor	0.571
6	Use information the doctor gives you to make decisions about your illness	0.559

Item no.	Scale items	Standardized factor loadings
1	I am reluctant about adopting new ways of doing things until I see them working for people around me. (Reverse)	0.648
2	I am receptive to new ideas.	0.646
3	I enjoy trying new ideas.	0.624
4	I consider myself to be creative and original in my thinking and behavior.	0.623
5	I find it stimulating to be original in my thinking and behavior.	0.618
6	I am an inventive kind of person.	0.606
7	I rarely trust new ideas until I can see whether the vast majority of people around me accept them. (Reverse)	0.602
8	I seek out new ways to do things.	0.601
9	I am aware that I am usually one of the last people in my group to accept something new. (Reverse)	0.574

Item no.	Scale items	Standardized factor loadings
1	Using medications belonging to family members is safe. (Reverse)	0.68
2	Using medications without a doctor’s advice is safe. (Reverse)	0.671
3	Only a doctor can decide on the necessity of medication.	0.646
4	Decisions regarding medication should only be made by a doctor.	0.636
5	Disregarding medication advice from anyone other than a doctor.	0.636
6	Trying to treat oneself with medications available at home. (Reverse)	0.629
7	Medications that work well for relatives will also work well for me. (Reverse)	0.618

The substantial reduction of items in the Individual Innovativeness Scale (IIS) and the Rational Drug Use (RDU) scale significantly enhances the psychometric quality and construct validity of the measurement models. By removing items with low factor loadings (*λ* < 0.50$), measurement noise and error variance were minimized, leading to a substantial improvement in the Average Variance Extracted (AVE) and establishing stronger convergent validity. Furthermore, pruning items with high cross-loadings or ambiguous phrasing strengthened discriminant validity and established clear boundaries between the latent constructs in the structural model. Importantly, the retained items maintain strong content coverage of the core theoretical domains: the 9 retained IIS items capture critical dimensions of innovativeness such as creativity, openness to new methods, and risk tolerance, while the 7 retained RDU items address essential behaviors including adherence to medical prescriptions, avoidance of self-medication, and reluctance to follow non-expert medical advice. Ultimately, this structural refinement yields a more simplified model that reduces respondent burden and response fatigue in practical applications without sacrificing diagnostic rigor.

Table 6 illustrates the bivariate correlations among the study variables. The results reveal a significant positive correlation between HL and RDU (*r* = 0.495**, *p* < 0.01**). Individual innovativeness is significantly and positively correlated with both HL (*r* = 0.315**, *p* < 0.01**) and AI attitude (*r* = 0.270**, *p* < 0.01**). However, the direct correlation between AI attitude and RDU is relatively weak (*r* = 0.046**, *p* > 0.05**). These initial correlation findings provide a solid foundation for testing the hypothesized structural paths.

**Table 6:** Correlation Matrix of the Study Constructs.

Variables	1	2	3	4
1. AI attitude	0.806			
2. HL	0.111*	0.837		
3. Individual innovativeness	0.270**	0.315**	0.854	
4. RDU	0.046	0.495**	0.106*	0.825

Diagonals in bold represent the square root of the Average Variance Extracted ( $\sqrt{\mathrm{AVE}}$ ) for each construct. Off-diagonal elements are bivariate correlations ( r ). **p < 0.01, *p < 0.05.

Table 7 presents the results of the structural equation modeling (SEM) and hypothesis testing. The structural path analysis reveals that individual innovativeness has a significant positive effect on HL (*β* = 0.308, *p* < 0.001) and RDU (*β* = 0.122, *p* = 0.018), providing support for H2. Furthermore, HL significantly impacts RDU (*β* = 0.201, *p* = 0.009), supporting H3. A favorable attitude toward AI significantly improves HL (*β* = 0.028, *p* = 0.005), thereby supporting H1. However, the direct path from AI attitude to RDU is not statistically significant (*β* = 0.008, *p* = 0.098).

**Table 7:** Structural path coefficients and hypothesis testing results.

Hypothesized path	Standardized beta (*β*)	*t*-value	*p*-value	95% bias-corrected CI [LLCI, ULCI]	Decision
Individual innovativeness → HL	0.308	6.54	0.001	–	Supported
Individual innovativeness → RDU	0.122	2.38	0.018	–	Supported
HL → RDU	0.201	3.40	0.001	–	Supported
AI attitude → HL	0.028	2.59	0.009	–	Supported
AI attitude → RDU	0.008	0.66	0.098	–	Not supported
AI attitude → HL → RDU	0.005	0.50	0.617	[−0.004, 0.018]	Not supported
Individual innovativeness → HL → RDU	0.025	3.55	0.002	[0.012, 0.045]	Supported

Regarding the mediation effects, the bootstrapping procedure demonstrated that HL significantly mediates the relationship between individual innovativeness and RDU (indirect effect *β* = 0.025, 95% CI [0.012, 0.045], *p* = 0.002), supporting H5. Conversely, the mediating role of HL between AI attitude and RDU was not statistically significant (indirect effect *β* = 0.005, 95% CI [−0.004, 0.018], *p* = 0.617), leading to the rejection of H4.

Discriminant validity was evaluated using the Fornell-Larcker criterion. As presented in Table 6, the square root of the AVE for each construct (bolded on the diagonal: 0.806 for AI Attitude, 0.837 for HL, 0.854 for Individual Innovativeness, and 0.825 for RDU) was strictly greater than its highest correlation with any other construct in the model. This confirms that each latent variable is empirically distinct and adequate discriminant validity is established.

### 3.1 Structural model and hypothesis testing

Before testing the structural relationships, the model’s overall goodness of fit was evaluated using established fit indices. The results demonstrated an acceptable fit to the empirical data: chi^2^/df = 2.45, RMSEA = 0.062, CFI = 0.935, TLI = 0.921, and SRMR = 0.058. After confirming the model fit, the hypothesized direct and indirect structural paths were analyzed.

The mediation hypotheses were tested utilizing a 5,000-resample bootstrapping method. A mediating effect is considered statistically significant if the 95% bias-corrected confidence interval (LLCI: Lower-Level Confidence Interval, ULCI: Upper-Level Confidence Interval) does not contain zero. As shown in the results, the indirect effect of HL was significant.

## 4 Discussion

### 4.1 Main findings and clinical implications

This study empirically investigates the complex relationships among attitudes toward AI, individual innovativeness, HL, and RDU within the digital health ecosystem. A pivotal finding of this research is that although a positive attitude toward AI significantly enhances HL (*β* = 0.028, *t* = 2.59, *p* = 0.005), it does not directly translate into RDU behavior (*β* = 0.008, *t* = 0.66, *p* = 0.098). This crucial gap between technological optimism and safe health outcomes is consistent with literature highlighting that positive technology adoption attitudes do not guarantee direct clinical compliance (31, 41). The empirical data suggest that while AI affinity broadens cognitive capacity by facilitating rapid access to health information, this informational advantage does not automatically translate into rational action requiring clinical discipline, which mirrors warnings that digital health search can lead to unverified self-treatment if not clinically filtered (14, 42). Conversely, individual innovativeness has a strong and significant impact on RDU, both directly (*β* = 0.122, *t* = 2.38, *p* = 0.018) and indirectly through HL (*β* = 0.025, 95% CI [0.012, 0.045], *p* = 0.002). This robust relationship reinforces the theoretical consensus that proactive innovative traits dynamically enhance health literacy, which in turn acts as a vital cognitive catalyst for safe medication management (12, 26). Ultimately, these findings demonstrate that, rather than a general interest in technological tools, an individual’s innovative disposition and proactive cognitive capacity critically shape rational health behaviors, positioning health literacy as a critical mediator in translating personal readiness into safe clinical habits.

When we further examine the clinical meaning of the findings, a phenomenon known as the ‘Digital Physician Illusion’ emerges, empirically highlighted by the non-significant direct (*β* = 0.008, *t* = 0.66, *p* = 0.098) and indirect (*β* = 0.005, 95% CI [−0.004, 0.018], *p* = 0.617) effects of AI attitudes on RDU in our structural model. Today, generative AI systems and large language models (LLMs) can generate the perception among users that they are consulting a medical expert (43). However, this situation triggers what is known in the literature as the ‘attitude-behavior gap’ (44). Although individuals accept the information they receive from AI as correct and believe their HL has increased, they do not put it into practice unless it undergoes clinical scrutiny. More alarmingly, as our non-significant pathways confirm, it can lead to uncontrolled drug use driven by a false sense of self-efficacy (44). The most vital academic distinction to be made here is the profound difference between the mere act of consuming medication and RDU, which is a strict clinical discipline requiring the use of the correct medication, at the optimal dosage, and at the appropriate time, always under the supervision of a physician or pharmacist (45). Although AI algorithms simplify clinical terminology and make information more accessible, they cannot replace a physician’s clinical judgment, ethical responsibility, or a pharmacist’s advisory role (46). Algorithms may push an individual toward drug consumption, but they cannot foster the discipline required to rationalize this consumption. At this point, the empathetic and guiding roles of physicians and pharmacists remain irreplaceable (47).

While general literature suggests that digital health nudges inherently improve treatment compliance (48, 49), our model significantly deviates by showing that attitudes toward AI do not predict RDU (*β* = 0.008, *t* = 0.66, *p* = 0.098). This discrepancy suggests that while positive AI attitudes significantly foster health literacy (*β* = 0.028, *t* = 2.59, *p* = 0.005), this informational acquisition fails to guarantee clinical discipline due to the prevalent risk of AI hallucinations (50, 51). Authoritative-sounding but clinically inaccurate AI outputs can create a false sense of comprehension (43, 52), where automation bias (53) prompts individuals to follow algorithmic advice without professional validation (54). Ultimately, this explains why HL does not significantly mediate the relationship between AI attitude and RDU (*β* = 0.005, 95% CI [−0.004, 0.018], *p* = 0.617), confirming that technological optimism cannot bypass the essential oversight of physicians or pharmacists (44, 47).

Our empirical results demonstrate that while individual innovativeness has a significant direct effect on RDU (*β* = 0.122, *t* = 2.38, *p* = 0.018), its indirect impact through HL is also statistically significant (indirect effect *β* = 0.025, 95% CI [0.012, 0.045], *p* = 0.002), supported by the highly significant paths from innovativeness to HL (*β* = 0.308, *t* = 6.54, *p* < 0.001) and HL to RDU (*β* = 0.201, *t* = 3.4, *p* = 0.009). This confirms that individual innovativeness alone does not promote sustainable behaviors such as RDU as effectively as it does when mediated by HL. Framed within Rogers’ Diffusion of Innovation theory (6), while individuals with innovative tendencies actively seek new information sources (55), our evidence confirms that HL must function as a vital cognitive catalyst to successfully translate this proactive disposition into rational clinical actions (15, 56). Ultimately, this synergy enables highly literate, innovative individuals to overcome digital information overload and effectively synthesise health data with clinical recommendations to make rational decisions (57).

The results provide critical policy understandings for stakeholders in the healthcare system. Given that our empirical model demonstrates that a favorable attitude toward AI does not directly translate into rational clinical behaviors (*β* = 0.008, *t* = 0.66, *p* = 0.098), physicians and pharmacists should adopt the role of ‘clinical navigators’, directing patients’ information-seeking behavior toward RDU rather than perceiving digital tools as professional threats. For healthcare administrators and policymakers, promoting the ‘human-in-the-loop’ model and strict interface standards as legal requirements for integrating AI applications in healthcare will be an essential safeguard against flawed algorithmic guidance (52). Furthermore, reflecting our evidence that technological adoption alone cannot replace professional clinical oversight, patients should be encouraged to view AI not as a substitute for a physician, but as a supplementary tool to enhance their understanding of health processes and facilitate professional consultation (44, 57).

Beyond the primary structural relationships, our demographic analyses provide critical insights into the underlying variations within the digital health ecosystem. The robust positive impact of higher educational attainment across all dimensions—health literacy, individual innovativeness, rational drug use, and AI acceptance—underscores that advanced information-evaluation skills are fundamental requirements for navigating digital health platforms safely. Furthermore, the higher AI adoption readiness observed among male participants aligns with existing technology acceptance literature regarding gender-based differences in digital affinity. Crucially, the finding that individuals without chronic illnesses exhibit significantly greater AI acceptance can be closely tied to the younger demographic profile of this cohort; their lower baseline healthcare anxiety likely fosters a more exploratory engagement with medical AI algorithms. Together, these demographic variations highlight that digital health interventions and AI integration strategies cannot follow a ‘one-size-fits-all’ approach, but must be tailored to the educational and demographic realities of the target population.

### 4.2 Theoretical implications

This study empirically examines the core relationships between attitudes toward AI, individual innovativeness, HL, and RDU in Northern Cyprus. The value of this study lies in its cross-disciplinary synthesis of the TAM and the HBM, creating a comprehensive framework for technology-oriented health behaviors (58, 59). By modeling how individual innovativeness and attitudes toward technology shape clinical behaviors, such as RDU, through HL, the study addresses a critical gap in the literature. Specifically, the empirical support for the positive pathways from individual innovativeness to both HL and RDU theoretically reinforces the role of proactive personality traits in driving health-related outcomes. Notably, the finding that HL serves a critical mediating function between individual innovativeness and RDU—acting as a ‘cognitive catalyst’—conceptually expands the Health Belief Model by positioning literacy as a vital cognitive tool that operationalizes self-efficacy and translates personal readiness into safe medication practices (60, 61). Conversely, the non-significant direct and indirect effects of AI attitudes on RDU theoretically demonstrate a key limitation in classical technology acceptance models within clinical contexts. This suggests that while technological optimism (TAM) successfully enhances cognitive capacity (HL), it fails to bypass the strict clinical discipline and risk-avoidance mechanisms (HBM) required for rational health behaviors when isolated from professional validation.

### 4.3 Practical implications

These findings reveal that while AI systems make medical information highly accessible—as demonstrated by our confirmed path between AI attitudes and health literacy—they are not a sufficient filter for translating this data into RDU, which inherently requires clinical discipline. The risks posed by potentially misleading algorithmic guidance underscore the persistent need for professional oversight (14). Crucially, our evidence that individual innovativeness and health literacy are the actual drivers of RDU shows that success in digital health transformation relies on human behavior and innovative engagement rather than advanced algorithms alone. Despite AI making information more accessible, physicians and pharmacists remain indispensable for rational clinical decision-making (62).

### 4.4 Limitations and future directions

This study must be interpreted considering certain methodological limitations. First, the cross-sectional design makes it impossible to prove direct cause-and-effect relationships, highlighting the need for future longitudinal studies to track the evolution of these dynamics, particularly to observe whether the mediating role of health literacy between individual innovativeness and rational drug use remains stable over time. Second, while the study provides a valuable snapshot of a specific digital and cultural ecosystem, the findings should be extrapolated to other contexts with caution, as cultural and demographic variables significantly influence technology acceptance and health behaviors. Third, reliance on self-reported survey data introduces the potential for response bias, common in behavioral research. Finally, given the rapid evolution of AI, future research should assess how more autonomous, ethically oriented, and empathic AI capabilities might reshape these attitudes and influence behavioral outcomes over time, particularly across different demographic groups. Additionally, because AI is evolving so quickly, our findings capture just one specific moment in time. Future longitudinal studies, especially those exploring more advanced AI systems, will be essential to better understand how technology continues to reshape HL, providing a dynamic perspective to evaluate whether long-term interaction with advanced AI can eventually bridge the non-significant attitude-behavior gap identified in our cross-sectional results. While AI simplifies access to information, professional guidance from physicians and pharmacists is needed for transforming information into safe treatment.

## 5 Conclusion

This study empirically investigates the complex relationships among attitudes toward AI, individual innovativeness, HL and RDU within the digital health ecosystem. The key finding is that while positive attitudes toward AI enhance HL, they do not directly translate into RDU behavior. By contrast, individual innovativeness significantly impacts RDU both directly and through the mediating role of HL. These findings highlight the “Digital Physician Illusion,” emphasizing that algorithmic guidance cannot replace the indispensable oversight of healthcare professionals. Moving forward, a “human-in-the-loop” approach that positions AI as a supplementary tool rather than a substitute for clinical judgment will be essential for fostering both HL and RDU behaviors in digital health ecosystems.

## Data Availability

The original contributions presented in the study are included in the article/Supplementary material, further inquiries can be directed to the corresponding author.
